# Development and validation of a survey on reproductive health behaviors to reduce exposure to endocrine-disrupting chemicals in Koreans

**DOI:** 10.3389/frph.2025.1519896

**Published:** 2025-03-14

**Authors:** Hye Jin Kim, Sung Hae Kim, So Young Choi, Gisoo Shin

**Affiliations:** ^1^ Department of Nursing, Changshin University, Gyeongsangnam-do, Republic of Korea; ^2^ Department of Nursing, Tongmyong University, Busan, Republic of Korea; ^3^ College of Nursing, Sustainable Health Research Institute, Gyeongsang National University, Jinju, Republic of Korea; ^4^ College of Nursing, Chung-Ang University, Seoul, Republic of Korea

**Keywords:** questionnaire, reproduction, health, behaviors, endocrine-disrupting chemicals, factors

## Abstract

**Introduction:**

Recently, issues related to climate change and endocrine-disrupting chemicals have come to the forefront. In particular, the pursuit of convenience has led to increased exposure to endocrine-disrupting chemicals in daily life, posing threats to reproductive health, including infertility and cancer. Therefore, this study aims to develop a questionnaire to assess the reproductive health behaviors of Koreans for reducing exposure to endocrine-disrupting chemicals, and to verify its reliability and validity.

**Materials and method:**

This methodological study involved 288 adult men and women in South Korea and conducted item analysis, exploratory factor analysis, and confirmatory factor analysis.

**Results:**

The developed survey questionnaire consists of four factors and 19 detailed items related to reproductive health behaviors and reproductive health promotion behaviors through the main exposure routes of endocrine-disrupting chemicals: food, respiratory pathways, and skin absorption.

**Conclusion:**

Based on the results of this study, it is hoped that research on reproductive health behaviors aimed at reducing EDC exposure will expand, considering various environments such as national and ethnic differences.

## 1 Introduction

Reproductive health is a holistic state of well-being that extends beyond the mere absence of disease or disorders related to the reproductive system. It encompasses physical, mental, and social well-being and includes the right to freely decide when and how many children to have (1). In South Korea, the concepts of reproduction and reproductive health began gaining recognition in 2009, covering health issues related to the reproductive system throughout the lifespan of both men and women (2). Reproductive health is closely linked to the country's declining birth rate, with infertility being one of the primary contributing factors (3). Also, reproductive health promotion activities include positive actions for reproductive health, such as safe practices and responsible sexual behavior, genital health management, prevention of sexually transmitted diseases, family planning for healthy pregnancy and childbirth, and genital hygiene management (4). Reproductive health and reproductive health practices are closely related to infertility, and this relationship is also interconnected with lifestyle choices (5). Recently, exposure to environmental hormones has emerged as a significant issue among these lifestyle factors (6). Environmental hormones, also known as endocrine-disrupting chemicals (EDCs), are synthetic chemicals (EDCs) are synthetic chemicals that have been in use since the industrialization period (7). Around 100 types of these EDCs are known to have harmful effects on human health, but the specific chemicals classified as environmental hormones vary by country and institution due to the absence of standardized criteria (8).

Some EDCs directly bind to hormone receptors, such as estrogen, androgen, and thyroid hormone receptors, either mimicking or blocking their functions, thereby disrupting the body's normal physiological processes (9). Moreover, the effects of EDC exposure vary depending on the stage of life-fetal development infancy, childhood, adolescence, or adulthood—and can differ by gender. Therefore, even if exposure levels fall within permissible limits, it is difficult to conclude that they are entirely safe, particularly for couples trying to conceive who may be more vulnerable to these chemicals (10). Additionally, current exposure limits for commonly used EDCs do not fully consider the nonlinear dose-response relationships or the diverse characteristics of different EDCs. As a result, even low levels of exposure can have adverse health effects under certain (11).

EDCs enter the body through various exposure routes, including food, air, and skin, making them nearly unavoidable in daily life (7–9). Studies indicate that the reproductive system is the most affected human system by EDC exposure, as many EDCs act like estrogen or have anti-estrogen effects. These effects can lead to reduced sperm count, smaller male reproductive organs, feminization of male reproductive traits, abnormal reproductive behaviors, and decreased fertility rates (8). Furthermore, increasing rates of prostate cancer, testicular cancer, breast cancer, infertility, and early puberty are suspected to be linked to cumulative EDC exposure (12).

Despite the known health risks of EDCs, public awareness of daily exposure sources remains low. This lack of awareness is primarily due to insufficient knowledge about prevention measures, as well as the prioritization of convenience in modern life, leading to desensitization to EDC exposure risks (13). In contrast, reproductive health behaviors aimed at reducing EDC exposure focus on minimizing risks by avoiding or limiting exposure in daily life (14).

Methods for measuring EDCs typically involve blood or urine analysis, but these methods may have limitations in terms of practicality, cost, or invasiveness in certain environments. As alternatives, non-invasive biomonitoring methods, such as hair or saliva samples, are being used, or EDC-related biomarkers are measured in the environment (e.g., air, water, food samples), or wearable devices are employed to track exposure over time, providing relevant data on EDC exposure without invasive procedures (15). Nevertheless, in order to comprehensively understand the impact of EDCs on reproductive health and to assess health behaviors for preventing EDC exposure in both men and women, a widely accessible survey questionnaire is needed. Thus, this study aims to define reproductive health behaviors aimed at reducing EDC exposure based on previous research and to develop a self-administered questionnaire for assessing the degree of EDC exposure in Korean participants.

## 2 Materials and methods

### 2.1 Research design

This study is a methodological investigation designed to develop and validate a survey questionnaire that assesses reproductive health behaviors aimed at reducing EDC exposure among adult men and women.

### 2.2 Participants

The participants were recruited from eight metropolitan cities in South Korea: Seoul, Busan, Daegu, Incheon, Gwangju, Daejeon, Ulsan, and Sejong. The sample distribution was based on the 2022 Korean population. For the validation of the questionnaire's validity, the required sample size should be at least five times the number of items, or ten times the number of items for stable validation. However, the appropriate sample size for factor analysis can vary depending on several conditions, and the adequacy of the sample size may depend on the level of communality of the variables. Additionally, when the communality of the sample is low, a sample size of 300–500 participants is sufficient. Based on these criteria, this study determined the sample size to be 330 participants, including a 10% dropout rate. After excluding 24 unreliable responses, data from 288 participants were analyzed.

### 2.3 Research procedures

#### 2.3.1 Initial item generation and development

The initial pool of items was developed through a review of existing survey questionnaire and relevant literature from 2000 to 2021. Based on this, we developed questions for each element based on the four reproductive health behavior factors to prevent general exposure to EDCs, focusing on exposure through the main routes of food, respiration, and skin. Items measuring reproductive health behaviors aimed at reducing EDC exposure in daily life were derived, resulting in 52 initial items. Examples include: “I often eat canned tuna,” “I use plastic water bottles or utensils,” and “I frequently dye or bleach my hair.”

#### 2.3.2 Content validity verification

A panel of five experts—including two chemical/environmental specialists, a physician, a nursing professor, and a Korean language expert—assessed content validity. The content validity index (CVI) for the 52 items was above .80, meeting the standard criteria. Four items were removed for failing to meet the required validity threshold, and others were revised based on expert feedback.

#### 2.3.3 Pilot study

A pilot study was conducted with ten adults (six women and four men) in Busan and Gyeongnam to identify items that were unclear or difficult to answer. Adjustments were made based on feedback regarding response time, item clarity, and questionnaire layout.

### 2.4 Measurement

The final survey questionnaire contained 19 items rated on a 5-point Likert scale (1 = strongly disagree to 5 = strongly agree). As a result of the final factor analysis, the four derived factors—health behaviors through food, health behaviors through breathing, health behaviors through skin, and health promotion behaviors—were named based on concepts that encapsulate the content of each factor's items. Higher scores indicated greater engagement in health behaviors to reduce EDC exposure. The reliability of this research questionnaire was measured using Cronbach's alpha, which was .80. This meets the verification criteria of at least .70 for a newly developed questionnaire and at least .80 for an established questionnaire.

### 2.5 Data collection

Data were collected between March 25 and April 27, 2023, at high-traffic areas such as train and bus terminals. Participants were recruited from Seoul (139 participants), Busan (46 participants), Daegu (33 participants), Incheon (33 participants), Gwangju (43 participants), Daejeon (20 participants), Ulsan (13 participants), and Sejong (3 participants), based on the distribution ratio applied to the 2022 Korean population. The researchers provided participants with papers explaining the purpose and details of the study and collected data from those who voluntarily agreed to participate. Survey completion took place at the collection site, and the completed surveys were sealed in collection envelopes and submitted to the researchers. The time required for survey completion was 15–20 min, and the participants were provided with small tokens of appreciation.

### 2.6 Data analysis

The data were analyzed using IBM SPSS Statistics 26.0 and IBM SPSS AMOS 23.0. First, descriptive statistics, such as means, standard deviations, frequencies, and percentages, were used to analyze the general characteristics of the participants. Second, the content validity was analyzed using an item-level content validity index (I-CVI). Third, item analysis, exploratory factor analysis (EFA), and confirmatory factor analysis (CFA) were conducted for construct validity verification. The item analysis involved calculating the mean, standard deviation, skewness, kurtosis, and item-total correlations. EFA included confirming the adequacy of the data through the Kaiser-Meyer-Olkin (KMO) and Bartlett's tests of sphericity. Principal component analysis was used to extract the factors, and varimax rotation was applied. Factors were selected based on eigenvalues greater than 1 and a scree plot examination, considering a cumulative explained variance of at least 50%. Item deletion was based on communalities and factor loadings, with items below .40 being removed. It was considered desirable to have at least three items per factor; therefore, factors with fewer than three items were excluded after item removal. A CFA was conducted to verify whether the model derived from the EFA adequately explained the factors. For CFA, items with standardized factor loading values below 0.40 were considered for deletion. The structural fit of the model was assessed using absolute fit indices [*χ*^2^ test, standardized root mean squared residual (SRMR), root mean square error of approximation (RMSEA)] and incremental fit indices (Comparative Fit Index (CFI), Tucker–Lewis Index (TLI), Parsimonious Comparative Fit Index (PCFI), and Parsimonious Normed of Fit Index (PNFI). Fourth, convergent validity was verified by calculating Pearson correlation coefficients for each domain. Finally, the reliability of the questionnaire was assessed using Cronbach's alpha.

### 2.7 Ethical considerations

Before conducting the study, approval was obtained from the Institutional Review Board (IRB No: 2023003. Participants were informed about the research purpose, duration, methods, and benefits. They were assured that there would be no disadvantages for declining participation or withdrawing consent after agreeing to participate. Additionally, participants were informed that they could withdraw from the study at any time if they experienced psychological discomfort during the survey process.

## 3 Results

### 3.1 Participant characteristics

The total number of participants was 288; their general characteristics are presented in Table 1. Of the 288 participants, 224 were women (77.8%) and 64 were men (22.2%), with a mean age of 39.27 ± 13.36 years. Among the respondents, 182 (63.2%) were married, and the highest level of education was a bachelor's degree (136 participants, 47.2%).

**Table 1:** General characteristics of participants (*N* = 288).

Characteristics	Categories	*N*	(%)
Gender	Men	62	(22.2)
	Women	224	(77.8)
Age (years)	<30	88	(30.6)
	30–39	55	(19.1)
	40–49	66	(22.9)
	50 ≤	79	(27.4)
Marital status	Single	106	(36.8)
	Married	182	(63.2)
Education	Under high school	67	(23.3)
	College	39	(13.5)
	University	136	(47.2)
	Over post graduate	46	(16.0)
Employed	Yes	155	(53.8)
	No	133	(46.2)
Experience of endocrine-disrupting chemicals exposure education	Yes	62	(21.5)
	No	226	(78.5)

### 3.2 Validity verification

#### 3.2.1 Item analysis and exploratory factor analysis

Results of the item analysis showed that the mean score of the items ranged from 2.08 to 3.47, with skewness values ranging from −1.67 to 0.34 and kurtosis values ranging from −1.74 to 3.45, all satisfying the normality criteria. The correlation coefficients between each item and the total score ranged from −.41 to .62. Fourteen items with correlation coefficients below .30 were deleted for further analysis.

#### 3.2.2 Exploratory factor analysis

An exploratory factor analysis was conducted using 34 items, excluding 14 items that were removed during item analysis. The KMO values ranged from .860 to .867, and Bartlett's test of sphericity yielded *χ*^2^ = 2,904.18 (*p* < .001), indicating suitability for factor analysis. EFA was conducted six times. The first analysis resulted in seven factors with eigenvalues greater than 1, but four items were deleted due to factor loadings below .40 and communalities below .40. Subsequent analyses resulted in six factors, and after further deletions, five factors with a total of 23 items were extracted, satisfying all criteria with factor loadings ranging from .75 to .52, communalities ranging from .67 to .42, and an explained variance of 54.11% (Table 2).

**Table 2:** Factor loadings of items according to the exploratory factor analysis.

Item	Factor loading					Communality
	1	2	3	4	5	
Q9	.751	.243	.029	.051	.025	.627
Q13	.742	.180	.176	−.023	.118	.628
Q10	.713	.065	.108	.022	.090	.533
Q4	.605	.141	−.103	.145	.181	.450
Q6	.585	−.006	.219	.401	−.056	.554
Q8	.516	.164	.337	.190	−.044	.445
Q29	.187	.742	.159	.226	.017	.662
Q30	.020	.716	.145	.370	−.032	.672
Q37	.248	.703	−.125	.020	.058	.576
Q5	.243	.575	.295	.109	.051	.492
Q26	.119	.542	.151	−.196	.337	.483
Q44	.116	.518	.315	.156	.282	.485
Q20	.054	−.054	.758	.034	.080	.588
Q21	.191	.101	.672	.147	−.074	.525
Q27	.005	.110	.653	.049	.151	.464
Q22	.008	.348	.574	−.049	.040	.454
Q17	.223	.144	.552	.197	.042	.416
Q41	.182	.194	−.034	.719	−.109	.601
Q43	.033	.116	.114	.658	.179	.492
Q42	.171	.142	.249	.593	.249	.525
Q47	.078	.176	−.065	.268	.748	.673
Q48	.039	−.061	.201	.456	.547	.553
Q11	.329	.180	.225	−.250	.541	.547
Eigen value	6.03	1.88	1.69	1.61	1.23	
% of variance	26.22	8.17	7.35	7.03	5.35	
Cumulative (%)	26.22	34.99	41.73	48.76	54.11	
KMO values				.867		
Bartlett's sphere formation test		*χ*^2^ = 1,884.27			df = 253	

#### 3.2.3 Confirmatory factor analysis

A confirmatory factor analysis was conducted to verify the construct validity of the final five-factor model, with 23 items derived from the EFA. The initial model fit indices were *χ*^2^ = 455.98 (*p* < .001), CMIN/df = 2.07, RMSEA = .06, SRMR = .08, GFI = .88, CFI = .86, TLI = .84, PCFI = .75, PNFI = .67. After deleting items with standardized coefficients below 0.5 and all items from the fifth factor, a modified model with four factors and 19 items was tested. The final model fit indices were *χ*^2^ = 269.48 *(p* < .001), CMIN/df = 1.85, RMSEA = .05, SRMR = .06, GFI = .91, CFI = .91, TLI = .90, PCFI = .78, PNFI = .71, meeting the adequacy criteria, and the TLI was close to .90. Therefore, the final model was confirmed (Table 3).

**Table 3:** Model fit of confirmatory factor analysis.

Fitness index	CMIN/df	RMSEA	SRMR	GFI	CFI	TLI	PCFI	PNFI
Criteria	≤3	≤.08	≤.05	≥.90	≥.90	≥.90	≥.50	.60∼.90
Model 1	2.07	.06	.08	.88	.86	.84	.75	.67
Model 2	1.85	.05	.06	.91	.91	.90	.78	.71

CFI, comparative fit index; CMIN/df, the ratio of chi-square to the degree of freedom; GFI, goodness-of-fit index; TLI, Tuckere-Lewis index; RMSEA, root mean square error of approximation; SRMR, standardized root mean squared residual; PCFI, parsimonious comparative fit index; PNFI, parsimonious normed-of-fit index.

#### 3.2.4 Convergent validity and discriminant validity

For convergent validity verification, standardized factor loadings of each item were examined, and if they were above .50 (*p* < .001), the composite reliability for each factor ranged from .78 to .91, exceeding .70, and the average variance extracted (AVE) ranged from .54 to .68, exceeding .50, indicating that convergent validity was established. For discriminant validity, the AVE values for each factor ranged from .54 to .68, and the squared inter-factor correlations (*Φ*2) ranged from .22 to .37, with AVE values greater than *Φ*2 values, indicating that the concepts between factors were distinct, thus confirming discriminant validity (Table 4).

**Table 4:** Construct validity based on confirmatory factor analysis of questionnaire.

Items	*β*	SE	C.R.	Factors				AVE	CR
				1	2	3	4		
Q4	.52	–	–	1				.60	.90
Q6	.59	.18	7.08						
Q8	.57	.17	6.91						
Q9	.72	.18	7.84						
Q10	.62	.18	7.28						
Q13	.73	.17	7.87						
Q5	.64	–	–	.561*	1			.68	.91
Q29	.78	.14	10.03						
Q30	.71	.13	9.47						
Q37	.56	.15	7.85						
Q44	.58	.13	8.14						
Q17	.59	–	–	.470*	.547*	1		.57	.87
Q20	.59	.18	7.07						
Q21	.66	.16	7.57						
Q22	.52	.16	6.55						
Q27	.54	.15	6.70						
Q41	.59	–	–	.497*	.608*	.463*	1	.54	.78
Q42	.66	.17	6.78						
Q43	.57	.21	6.43						

AVE, average variance extracted; CR, construct reliability; C.R., critical ratio; SE, standard error.

* *p* < .001.

#### 3.2.5 The naming of factors and the corresponding items

In naming the factors, it was determined that a higher factor loading indicates a better explanation of the factor. The name of each factor was given after identifying the common meaning of the items with high factor loadings. The first factor was named “Health behaviors through food,” and it includes items such as enjoying fast food more than twice a week, enjoying drinks served in hot cans or plastic containers, avoiding the use of plastic utensils when eating food, not using containers that are deformed or damaged, removing the wrap before using the microwave, and thoroughly washing vegetables and fruits before eating.

The second factor was named “Health behaviors breathing,” and it includes items such as ventilating the air when cooking food, ventilating the indoor air twice a day for at least 20 min, ensuring adequate ventilation when using paint or paint-like substances, not using air fresheners containing environmental hormones, and avoiding touching the nose with hands contaminated with harmful substances.

The third factor was named “Health behaviors through skin,” and the items in this factor include washing laundry together if possible, disposing of paper receipts containing BPA immediately, using soap or shampoo containing natural ingredients instead of synthetic detergents, safely disposing of waste containing mercury such as used batteries, and minimizing direct skin contact with coated paper when bleaching.

The fourth factor was named “Health promotion behaviors,” and the items under this factor include enjoying eating vegetables that help eliminate environmental hormones, checking for environmental hormone components in the products used, and having a habit of ventilating the air.

#### 3.2.6 Reliability verification

To verify the internal consistency reliability of the health behaviors questionnaire to reduce exposure to EDCs developed in this study, Cronbach's *α* values were calculated. The overall reliability of the questionnaire was .85, and the reliability of each sub-factor ranged from .63 to .79, confirming internal consistency (Table 5).

**Table 5:** Internal consistency reliability and split-half reliability of questionnaire.

Factor	Mean ± SD	Number of items	Cronbach's *α*	Spearman Brown coefficient
1. Health behaviors through food	3.07 ± 0.46	6	.79	.77
2. Health behaviors breathing	3.16 ± 0.43	5	.78	.74
3. Health behaviors through skin	2.79 ± 0.23	5	.71	.65
4. Health promotion behaviors	2.72 ± 0.52	3	.63	.63
Total	2.93 ± 0.34	19	.85	.71

EDCs, endocrine disrupting chemicals.

## 4 Discussion

This study developed a survey questionnaire to measure reproductive health behaviors aimed at reducing exposure to EDCs among Korean adults and tested its validity and reliability. The findings indicate that the survey questionnaire consists of four factors and 19 items, classified according to the routes of EDC exposure in daily life. This discussion focuses on reproductive health behaviors with an emphasis on these exposure pathways.

The four factors (1 = health behaviors through food, 2 = health behaviors breathing, 3 = health behaviors through skin, 4 = health promotion behaviors) identified in this study suggest that EDC exposure occurs naturally through daily activities. Notably, exposure via food was the most significant, reflecting modern dietary habits (7–9). Common behaviors contributing to EDC exposure include consuming fast too, using hot cans or plastic containers, employing plastic utensils, microwaving food with plastic wrap, and failing to wash fruits and vegetables thoroughly. These practices expose individuals to EDCs such as bisphenol-A (BPA), phthalates, and phenols, which have been linked to reproductive system disorders (14). In particular, the use of plastic containers and plastic wrap for reheating refrigerated or frozen foods has been associated with EDC exposure and an increased risk of reproductive system cancers (16). Additionally, polystyrene cups, commonly used for instant cup noodles, release styrene dimers and trimers when exposed to hot water (17). Substances such as BPA, which are EDCs, are used to prevent corrosion inside canned goods. These substances can decompose from the container into the food stored inside the can, depending on storage conditions and temperature. Particularly, it has been proven through numerous studies that the concentration of EDCs is higher in canned foods compared to fresh foods (18). In South Korea, previous research found that BPA, a representative EDC, was detected in 21 out of 25 canned food products (19).

According to previous studies, one reason for the increasing rate of polyciticovarysyndrome (PCOS) in South Korea is the change in lifestyle. In particular, the rise in the consumption of cup noodles and canned foods has been linked to the increase in PCOS, as evidence of a connection between BPA and PCOS was found in systematic reviews and meta-analyses (20). Despite these risks, the food industry continues to use polystyrene due to its processing advantages and cost-effectiveness (8). Raising public awareness of EDC exposure through food should be a priority, and educational initiatives promoting safer consumption practices are essential.

The second factor identified in this study is related to behaviors that reduce respiratory exposure, including the use of fabric softeners and air fresheners, the importance of indoor air ventilation, and touching the nose with contaminated hands. Many fabric softeners and air fresheners contain antimicrobial and antifungal agents, including benzisothiazolinone, as well as quaternary ammonium compounds (21). Spray products pose a greater health risk than liquid or gel alternatives due to their potential for inhalation. Although formaldehyde levels in spray fabric deodorizers and air fresheners are lower than in liquid or gel products, they still present a significant health risk (22). Inhaled endocrine disruptors can lead to precocious puberty in children and reproductive disorders in fetuses, as their natural defense and metabolic systems are not fully developed. These effects may remain latent through childhood and manifest later as reproductive dysfunction or obesity in adulthood. Proper ventilation is essential in mitigating these risks (23), and public education should emphasize the importance of avoiding nasal contact with contaminated surfaces.

The third factor concerns behaviors that reduce skin absorption of EDCs, including the use of synthetic detergents, shampoos, soaps, mercury-containing products, and coated or heat-transfer papers. The skin serves as a vital barrier against physical or chemical damage, yet direct contact with EDCs can compromise its integrity (24). These chemicals affect various skin cells-including keratinocytes, follicle stem cells, sebaceous cells, melanocytes, and fibroblasts-as well as immune cells like such as T lymphocytes and Langerhans cells, leading to conditions like skin cancer, inflammatory and allergic skin diseases, acne, pigmentation disorders, and premature aging (25). Surfactants in shampoos, soaps, and shower products are easily absorbed through the skin and are not readily excreted; in women, they accumulate in the reproductive system, while in men, they build up in the kidneys, increasing the risk of infertility, breast cancer, and prostate cancer (26). Although there is a growing interest in natural and plant-based alternatives, synthetic detergents, remain widely used due to their convenience (25). Therefore, further research is needed to elucidate the precise mechanisms of EDC-related reproductive disorders via skin absorption. Additionally, public awareness campaigns should emphasize the risks associated with EDC skin exposure.

The final factor involves behaviors that promote reproductive health, such as consuming fiber-rich green vegetables to aid in EDC elimination, ensuring regular indoor ventilation, and developing the habit of checking for harmful substances in consumer products. Although concerns about EDC exposure have gained attention (8, 13), standardized guidelines for identifying EDCs and assessing their risks remain lacking. This is due to the complexity of EDC testing in real-life environments, differences in administrative procedures across countries, and the continuous emergence of new EDCs each year (8). Therefore, maintaining awareness of EDC exposure and adopting health-promoting behaviors are essential. Notably, it takes approximately three to five years for the reproductive system to eliminate half of the accumulated EDCs (27), underscoring the importance of long-term behavioral changes in reducing the risk of reproductive diseases. Additionally, previous meta-analysis studies on the impact of EDC exposure by gender (28) reported that no consistent evidence was found regarding the association between EDC exposure and its effects. In other words, it has not been possible to identify specific prenatal or postnatal exposure periods that are particularly critical or vulnerable to EDCs. This is influenced by various methodological issues and inconsistencies, and the evidence for specific exposure-outcome associations remains insufficient. Therefore, standardized research methods for EDC exposure are needed, and large-scale longitudinal epidemiological studies are required in the future to clarify overall associations.

## 5 Conclusion

This study developed a questionnaire to assess reproductive health behaviors aimed at reducing EDC exposure among Korean adults and validated its reliability. The survey questionnaire is applicable to both genders, emphasizing the importance of reproductive health behaviors in minimizing EDC exposure. However, a limitation of this study is that participants were recruited through convenience sampling, and furthermore, the majority of participants were women rather than men. This highlights the need for future research using more diverse and representative samples. Additionally, considering the differences in living environments between countries and ethnic groups based on gender differences, future studies should take these differences into account. The findings of this study contribute to raising awareness of reproductive health behaviors that can help reduce EDC exposure, ultimately promoting better health outcomes.

## Data Availability

The original contributions presented in the study are included in the article/Supplementary Material, further inquiries can be directed to the corresponding author.

## Appendix

Survey questionnaire for reproductive health behaviors to reduce endocrine-disrupting chemicals.

**Table:**

Factors	Items structure	Cronbach's α
Health behaviors through food	I enjoy eating fast food more than twice a week.^a^	.79
	Enjoy drinks served in hot cans or plastic containers.^a^	
	Avoid using plastic utensils when eating food.	
	Do not use containers that are deformed or damaged.	
	When using the microwave, remove the wrap before use.	
	Wash vegetables and fruits thoroughly before eating.	
Health behaviors breathing	Ventilate when cooking food.	.78
	Ventilate the indoor air twice a day for at least 20 min.	
	If you use paint (paint-like substances), ensure adequate ventilation.	
	Do not use air fresheners containing environmental hormones.	
	Do not touch your nose with hands contaminated with harmful substances.	
Health behaviors through skin	Wash laundry together if possible.	.71
	Dispose of paper receipts containing BPA immediately.	
	Use soap or shampoo containing natural ingredients rather than synthetic detergents.	
	Safely dispose of waste containing mercury, such as used batteries	
	Bleaching, minimizes direct skin contact with coated paper.	
Health promotion behaviors	Enjoy eating vegetables that help eliminate environmental hormones.	.63
	Check for environmental hormone components in the products you use.	
	Have a habit of ventilating the air	

^a^ Reverse question.
